# Deep learning model shows pathologist-level detection of sentinel node metastasis of melanoma and intra-nodal nevi on whole slide images

**DOI:** 10.3389/fmed.2024.1418013

**Published:** 2024-08-22

**Authors:** Jan Siarov, Angelica Siarov, Darshan Kumar, John Paoli, Johan Mölne, Noora Neittaanmäki

**Affiliations:** ^1^Department of Laboratory Medicine, Institute of Biomedicine, Sahlgrenska Academy, University of Gothenburg, Gothenburg, Sweden; ^2^Department of Clinical Pathology, Sahlgrenska University Hospital, Region Västra Götaland, Gothenburg, Sweden; ^3^Aiforia Technologies Plc., Helsinki, Finland; ^4^Department of Dermatology, Institute of Clinical Sciences, Sahlgrenska Academy, University of Gothenburg, Gothenburg, Sweden; ^5^Department of Dermatology and Venereology, Sahlgrenska University Hospital, Region Västra Götaland, Gothenburg, Sweden

**Keywords:** deep learning, artificial intelligence, digital pathology, dermatopathology, sentinel node biopsy, nodal melanoma metastasis, intra-nodal nevus deep learning, intra-nodal nevus

## Abstract

**Introduction:**

Nodal metastasis (NM) in sentinel node biopsies (SNB) is crucial for melanoma staging. However, an intra-nodal nevus (INN) may often be misclassified as NM, leading to potential misdiagnosis and incorrect staging. There is high discordance among pathologists in assessing SNB positivity, which may lead to false staging. Digital whole slide imaging offers the potential for implementing artificial intelligence (AI) in digital pathology. In this study, we assessed the capability of AI to detect NM and INN in SNBs.

**Methods:**

A total of 485 hematoxylin and eosin whole slide images (WSIs), including NM and INN from 196 SNBs, were collected and divided into training (279 WSIs), validation (89 WSIs), and test sets (117 WSIs). A deep learning model was trained with 5,956 manual pixel-wise annotations. The AI and three blinded dermatopathologists assessed the test set, with immunohistochemistry serving as the reference standard.

**Results:**

The AI model showed excellent performance with an area under the curve receiver operating characteristic (AUC) of 0.965 for detecting NM. In comparison, the AUC for NM detection among dermatopathologists ranged between 0.94 and 0.98. For the detection of INN, the AUC was lower for both AI (0.781) and dermatopathologists (range of 0.63–0.79).

**Discussion:**

In conclusion, the deep learning AI model showed excellent accuracy in detecting NM, achieving dermatopathologist-level performance in detecting both NM and INN. Importantly, the AI model showed the potential to differentiate between these two entities. However, further validation is warranted.

## 1 Introduction

Melanoma is one of the deadliest skin cancers, with its prognosis closely linked to the invasion depth and presence of nodal metastasis (NM) at the time of diagnosis. The American Joint Committee on Cancer (AJCC) staging system assesses melanoma using three parameters: T (primary tumor), N (regional lymph nodes), and M (distal metastases). Sentinel lymph node biopsy (SNB), which involves the excision and histopathological examination of the first draining lymph node, has become an established procedure for identifying NM. Accurate melanoma staging is crucial for evaluating prognosis and planning appropriate treatment. The prognosis for stage III melanoma patients is significantly worse compared to those in stage I or II, who do not have metastatic disease.

Furthermore, the number of metastatic lymph nodes significantly affects prognosis (1). Therefore, accurate SNB status is a crucial part of melanoma staging. Patients with metastases found in the SNB are classified as stage III, where adjuvant treatments with targeted therapies (BRAF and MEK-inhibitors) and immunotherapy are often considered (2). However, even patients in stage IIB or IIC without verified metastatic disease benefit from adjuvant treatments (3).

SNBs show NM in 6–24% of cases (4). However, small metastatic cell aggregates and isolated tumor cells (≤ 0.2mm) can easily be missed (5). Additionally, benign intra-nodal nevus (INN) is found in 1–24% of SNBs, and it can co-exist with NM and may be misclassified as such (6–8). INNs are most commonly found in SNBs of the neck and are associated with a high number of cutaneous nevi (9). The mutation profiles of INN and NM differ, indicating that INNs descend from previously UV-exposed BRAF wildtype cutaneous melanocytes rather than from primary MM or arrested progenitor cells (10). It is important to differentiate between NM and INN since patients with INN in SNBs have a prognosis similar to that of patients with negative SNBs and, therefore, do not require additional therapy (11).

There is high discordance in assessing SNB positivity, which may lead to false staging and inadequate use of adjuvant therapies (12). Immunohistochemistry (IHC) is used to aid the detection of NM and to differentiate between NM and INN (13). Thus, several tissue slides, including hematoxylin-eosin (H&E) and IHC, are prepared for each excised lymph node. This makes the laboratory process and interpretation of SNBs in melanoma patients time consuming. The time-consuming process of evaluating histopathological SNB slides, combined with an increasing number of samples, delays diagnosis and increases costs (14, 15). Taking into consideration the increasing workload, new solutions are warranted.

Digital whole slide imaging enables the implementation of artificial intelligence (AI) in digital pathology. Deep learning, in particular, has enabled rapid advances in computational pathology (16, 17). It has been shown that the use of deep learning with convolutional neural networks (CNN) enables the automatic detection of lymph node metastases in breast cancers, gastric cancer, and colorectal cancers on H&E-stained slides (18–21). AI has also shown potential in melanoma diagnosis (22). Interestingly, CNN algorithms appear capable of making decisions based solely on H&E-stained slides and even outperform pathologists (23). CNNs are advanced mathematical models that can handle the complexity of H&E. In contrast, traditional handcrafted algorithms, such as thresholding for image analysis, are more simplistic and depend on a very distinct contrast between colors (24). Thus, the latter has predominantly been useful for IHC slides in pathology.

Furthermore, AI has been studied to predict the SNB status from primary melanomas (25). A deep learning method in one recently published study could identify NM in SNB sections (2). However, studies regarding INN detection are still rare. To the best of our knowledge, no published studies regarding automated AI-based solutions for melanoma NM and INN morphologic detection exist.

Our study aimed to compare the capability of AI and dermatopathologists in detecting NM and INN in SNBs.

## 2 Methods

The study protocol followed the Declaration of Helsinki. According to the Swedish Ethical Review Authority (Dnr 2022-02165-01), the law states that our project did not require ethical approval or informed patient consent because the material is completely anonymized.

### 2.1 Dataset

We retrospectively collected 501 H&E-stained whole slide images (WSIs), including NM and INN, from 207 SNBs diagnosed at the Department of Pathology at Sahlgrenska University Hospital (Gothenburg, Sweden) between 2017 and 2020. The inclusion criteria required that both the H&E slides and IHC stainings from the same slides be available. All the included cases were formalin fixed and paraffin embedded. The glass slides were anonymized manually with a case number and then digitally scanned for whole slide imaging in 40x mode (0.23 μm/pixel, 20x objective lens) using the Nanozoomer Digital Slide Scanner S210 (NDP), Hamamatsu Photonics K.K., Shizuoka, Japan. After scanning, 16 WSIs (from 11 SNBs) were excluded due to poor image quality. Therefore, 485 WSIs from 196 SNBs were included in the study.

The dataset was randomly divided into training, validation, and test sets (Table 1). In cases where several slides were from the same SNB, they were included in the same dataset to ensure that slides from the same tumor did not end up in both the training and test sets. The microanatomical location of the NM is shown in Supplementary Table S1.

**Table 1:** The number of cases and whole slide images included in the training, validation, and test sets.

	**Cases**	**WSIs**
Training set	125	279
NM	25	66
INN	14	60
NM+INN	5	12
Tumor-free	48	141
Validation set	47	89
NM	17	20
INN	9	9
NM+INN	1	2
Tumor-free	20	58
Test set	61	117
NM	15	27
INN	6	9
NM+INN	0	0
Tumor-free	40	81
Total	196	485

WSIs, whole slide images; NM, nodal metastasis; INN, intra-nodal nevus.

### 2.2 Reference standard

IHC slides, including stainings with SOX10 (AVI 3099G, Ready-to-Use, Clone BC34, Mouse Monoclonal Primary Antibody, BioCare Pacheco, CA, USA) and HMB45 (Dako FLEX Monoclonal Mouse Anti-Human Melanosome, Clone HMB45, Ready-to-Use, Dako Omnis, Agilent, CA, USA), were collected for all 494 corresponding H&E slides. Along with cell morphology, these served as the reference standard for detecting NM (SOX10+, HMB45+) and INN (SOX10+, HMB45-). The cell morphology supporting NM diagnosis included larger cell size, a higher nuclear-to-cytoplasmic ratio, more prominent nucleoli, and mitotic figures. In the cases of INN, the cells are typically smaller than NMs and uniform, with limited cytoplasm and minimal cytoplasmic melanin. The nuclei of INN are unremarkable, lacking prominent nucleoli and mitotic figures (26). An experienced dermatopathologist reassessed all the slides for the study.

### 2.3 Annotations

The training set consisted of 279 WSIs, including 66 with NM, 60 with INN, 12 with both NM and INN, and 141 with tumor-free lymph nodes. Annotations were performed only on training set WSIs, while the validation set and test set WSIs remained unannotated. The annotations were performed manually by an experienced dermatopathologist with the help of IHC using corresponding serial slides (HE, SOX10, HMB45, HE). In total, 5,956 training regions were used. The annotations were made in two “layers”: first, teaching the AI model to recognize the tissue and, afterward, to recognize the tumor regions within the tissue.

**Figure 1:**
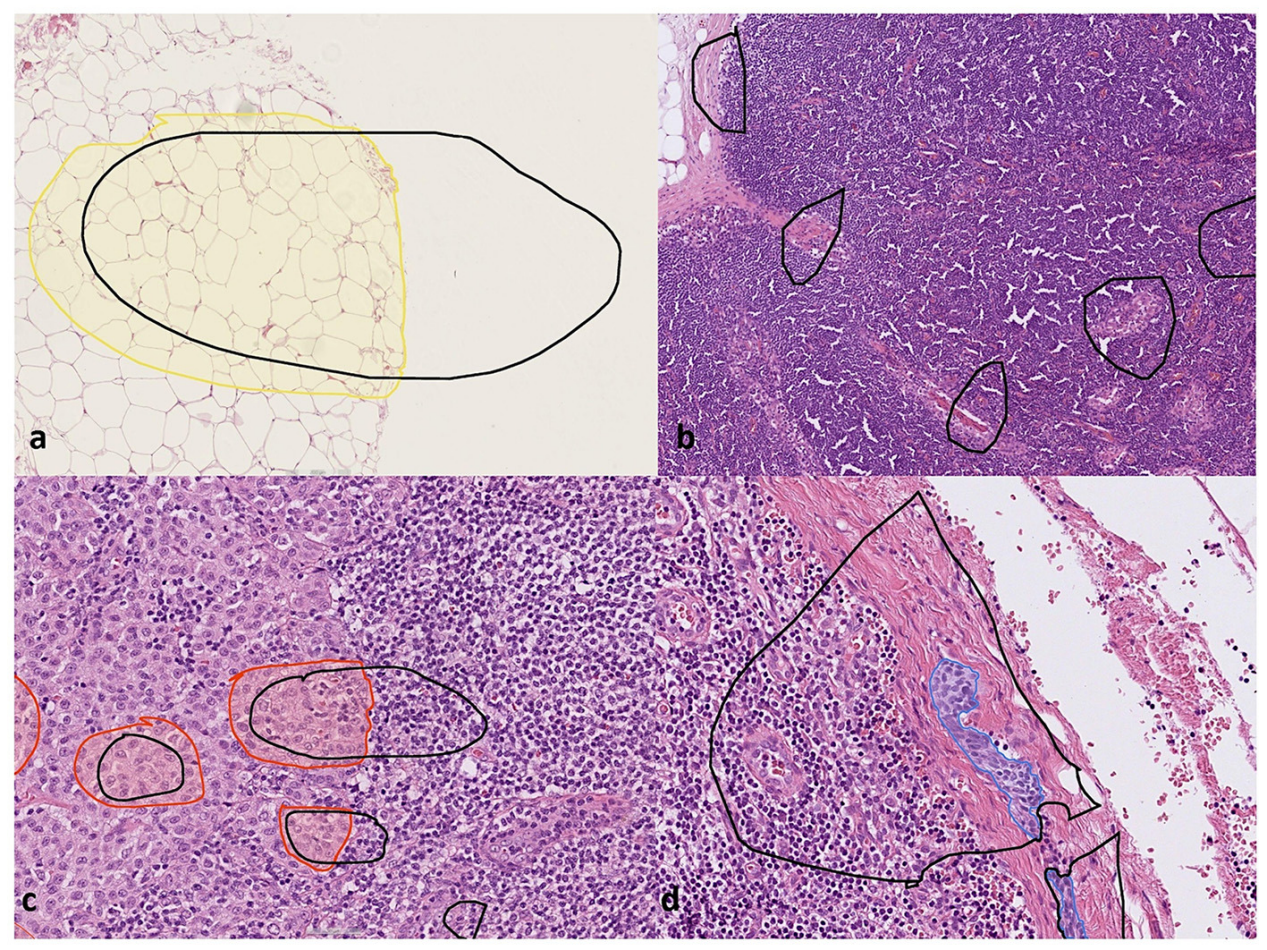
Training the deep learning artificial intelligence model. The areas marked in black correspond to training regions, and the areas marked with colors correspond to different annotated layers within the training regions: **(a)** tissue annotation (yellow). The white unannotated region in tissue annotation was trained not to be detected by the AI model, **(b)** areas with background tissue marked as “empty” training regions, **(c)** nodal melanoma metastasis annotation (red), and **(d)** intranodal nevus annotation (blue). Furthermore, the unannotated empty areas within the training regions in **(c, d)** were included in the training as background.

The different annotated layers within the training regions were named a “tissue layer,” with annotations representing the overall tissue areas on the slides, and a “tumor layer,” with annotations for areas with NM, INN, and background (lymphatic and connective tissue) (Figure 1). The total annotated areas were 213.01 mm^2^ for the tissue layer (approximately one annotation per slide) and 1,950.85 mm^2^ for the tumor layer (mean of seven annotations per slide). In total, 18.65 mm^2^ of the area was annotated as NM, and 0.56 mm^2^ was used for INN. The goal was to include as many heterogeneous areas as possible. Isolated tumor cells, when found, were also annotated in the tumor layer. Tissue artifacts were annotated as background.

### 2.4 Training the AI model

The AI model was generated using Aiforia Create (Aiforia Create Version 5.3, Aiforia Technologies Plc, Helsinki, Finland), a commercial image management and analysis cloud platform that facilitates the development of a machine learning model with CNN and supervised learning. The deep CNNs were trained using a supervised method, where pixel-level segments provided the annotations. The trained CNNs performed semantic segmentation at the pixel level, predicting each pixel to belong to either the foreground or background class. Segmentation-based outcome prediction was performed using semantic segmentation with defined complexity.

An expert pathologist provided fully supervised pixel-level input using IHC. The CNN used is based on U-net architecture. The created AI model used a parent–child architecture with two layers: the tissue layer as the “parent layer” and the tumor layer as the “child layer”. These layers were independent neural networks arranged hierarchically, with the child clipped by pixels to wherever the parent (i.e., tissue) was detected. The tissue layer was trained to identify high-quality tissue areas (tissue layer) and exclude tissue artifacts, while the tumor layer was trained to detect NM and INN according to the annotations. This configuration resulted in one combined AI model that first performs quality control on tissue architecture, followed by tumor detection within the high-quality tissue area in one analysis run, instead of two independent analyses. Aiforia Classic Default Neural Networks were used both for tissue and tumor layers.

A total of eight training sessions were conducted to create the final AI model. After each training session, additional annotations were made in the training set WSIs. Depending on the layer complexity, the trainings were terminated prematurely if the learning curve was not steep enough. This could be directly observed by the training loss function. A lower error percentage could be expected if the training loss was minimal after all the training rounds were completed, which is generally defined by a non-significant increase in the training curve.

The final training was performed for 20,000 iterations, with 5,900 iterations (training loss: 0.0009) for the tissue layer and 13,320 iterations (training loss: 0.0089) for the tumor layer. In total, 5,956 training regions were used, and verifications yielded an error of 0.04% for the tissue layer (F1 Score: 99.98%) and 0.06% for the tumor layer (F1 Score: 97.04%).

Patches used in training and inference were extracted from the image pyramid at a level dependent on the field of view (region layer) or object size (object layer). The inference results were combined across patches and projected to other zoom levels, including the highest zoom level (i.e., slide level) for results. Morphometric analysis was enabled for the AI model before the image analysis run. Image analysis was conducted in the Aiforia Hub for WSIs in multiple batches.

Data were visually monitored, and pixel-level segmentation outcomes were generated using Microsoft Excel. Image-level data were visualized on the user interphase under pixel information. The patch-level information was extrapolated to slide level with the help of aggregated pixel-level segmentation outcomes. For all the islands of semantic segments, the field of view, as deemed best by the domain expert depending on the feature of interest, was benchmarked to convert to a slide-level outcome. Every outcome was accompanied by a confidence outcome that directly represented or reflected the input user's confidence levels during the training process. A class confidence of 80% was set as a minimum threshold for areas of NM and INN. The detailed training and image augmentation parameters of the CNN models are shown in Supplementary Table S2.

### 2.5 Validating and testing the AI model

The performance of the AI model was validated on an unannotated set of WSIs using pixel-wise validation, which is explained in detail in the Supplementary material. After validation, the AI model was further trained to enhance its performance before running the test set analysis on a separate test set.

The algorithm's performance was compared against that of three individual dermatopathologists and the reference standard. The test set included 117 WSIs, comprising 27 with NM, 9 with NN, and 81 tumor-free lymph nodes. Three dermatopathologists, blinded to the AI results, each other's assessments, and the original pathology reports, independently evaluated the test set WSIs for the presence of NM, INN, or both. Using WSIs, dermatopathologists used variable digital magnification to evaluate the slides. They had access only to the H&E slides and no IHC slides. No time constraints were applied. The results were reported on a slide level (no tumor, NM, INN, or both) without marking the tumor regions. Additionally, for each slide, the dermatopathologists reported their confidence level on a 5-level scale (1 = very certain, 2 = moderately certain, 3 = average certainty, 4 = moderately uncertain, 5 = very uncertain).

### 2.6 Statistical analysis

No prior in-house data regarding the performance of an AI algorithm for this task were available. Therefore, no power analysis was conducted to predetermine an appropriate sample size. Instead, we aimed for a sample size similar to that used in a previous study for detecting breast cancer metastases (15). All data were analyzed using R version 3.5.3 (https://www.r-project.org/) with the assistance of a trained statistician.

Receiver operating characteristic (ROC) curves were created using the AI model with multiple gain settings. The gain setting controls the CNN's sensitivity to predict pixels belonging to a feature class. As the gain increases, the CNN becomes more sensitive, classifying more pixels into the designated feature class. These gain settings are specific to individual CNNs. In the ROC analysis, the gain setting was systematically adjusted to titrate the sensitivity of the AI model as a function of specificity. The gains ranged from 0.001 to 10 for both classes (INN and NM) and were incremented accordingly. Independent ROC curves were generated for the AI model with varying gain settings and plotted for visualization, with sensitivity on the Y axis and 1-specificity (false positive rate) on the X axis.

In addition, confusion matrices were generated to compare the AI model's performance with that of the dermatopathologists, calculating sensitivity, specificity, accuracy, and the respective *P*-value for each individual (using the exact binomial test, McNemar's test) (27). The 5-level pathologist confidence scale (1 being very certain and 5 being very uncertain of the asked diagnosis) was then used to obtain ROC curves. This scale was converted into a 10-grade scale from 0 to 1, with 0 indicating high certainty that the diagnosis is incorrect and 1 indicating high certainty that the diagnosis is correct.

A false-negative result was defined as either failing to identify the tumor or incorrectly labeling the tumor type. Therefore, a false-negative interpretation of a slide with NM included the results “INN” and “normal lymph node.” Similarly, a false-negative interpretation of an INN slide included the results “NM” and “normal lymph node.” Notably, no slides in the test set included both INN and NM on the same slide. Conversely, a false positive result was defined as either incorrectly labeling the tumor type or identifying healthy background tissue as a tumor.

## 3 Results

**Figure 2:**
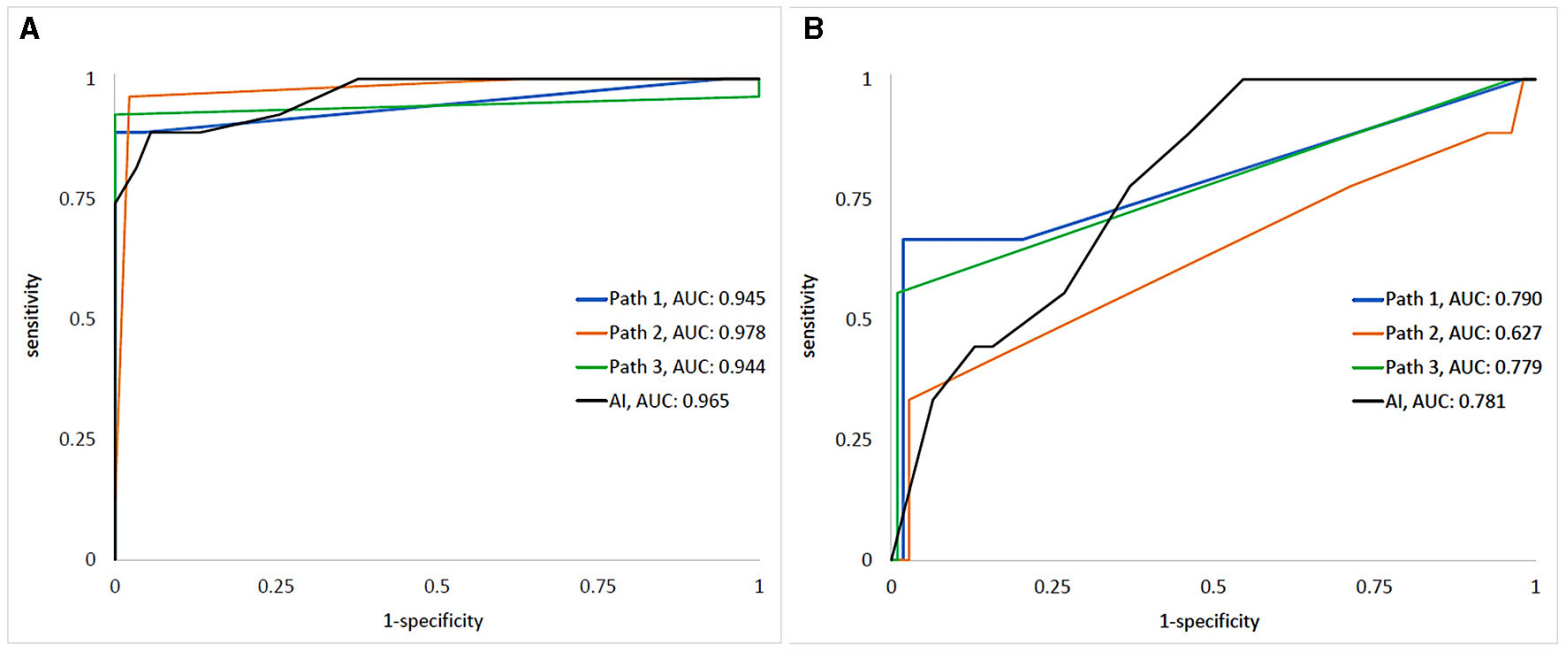
The ROCs/AUCs of the AI and dermatopathologists vs. the ground truth for detecting **(A)** melanoma metastases and **(B)** intra-nodal nevi. “Path 1–3” represents the human dermatopathologists, and “AI” represents the algorithm.

The AI model showed excellent performance with an area under the ROC (AUC) of 0.97 (95% CI: 0.94–0.98) for detecting NM, compared to the ground truth. For comparison, the AUC for individual dermatopathologists in NM detection ranged between 0.94 (95% CI: 0.88–1.00) and 0.98 (95% CI: 0.95–1.00). The AUC values for the AI and the individual dermatopathologists versus the ground truth are shown in Figure 2.

For the detection of INN, AUC was lower for both AI [0.78 (95% CI: 0.70-0.85)] and the dermatopathologists [range: 0.63 (95% CI: 0.42–0.83)−0.79 (95% CI: 0.61–0.97)].

The sensitivity for predicting NM was 89% (95% CI: 71–98%), 93% (95% CI: 76–99%), and 96% (95% CI: 81–100%) for the dermatopathologists compared to the AI's sensitivity of 89% (95% CI: 71–98%) (*p* = 1, *p* = 1, *p* = 0.63, respectively). The specificity was 98% (95% CI: 92–100%), 100% (95% CI: 96–100%), and 100% (95% CI: 96–100%) for the dermatopathologists compared to 94% (95% CI: 88–98%) for the AI (*p* = 0.45, *p* = 0.063, *p* = 0.063, respectively). Youden's index for predicting NM for the dermatopathologists was 0.89 (95% CI: 0.71–0.98), 0.93 (95% CI: 0.76–0.99), and 0.94 (95% CI: 0.79–0.98), compared to the AI's index of 0.83 (95% CI: 0.65–0.92).

The sensitivity for predicting INN was 33% (95% CI: 8–70%), 56% (95% CI: 21–86%), and 67% (95% CI: 30–93%) for the dermatopathologists compared to the AI's sensitivity of 78% (95% CI: 40-97%) (*p* = 0.008, *p* = 0.31, *p* = 0.73, respectively). The specificity was 97% (95% CI: 92–99%), 98% (95% CI: 94–99%), and 99% (95% CI: 95–100%) for the dermatopathologists compared to 63% (95% CI:53–72% for the AI (*p* < 0.001, *p* < 0.001, *p* < 0.001, respectively). Youden's index for predicting INN for the dermatopathologists was 0.31 (95% CI: 0.047–0.67), 0.55 (95% CI: 0.20–0.85), and 0.65 (95% CI: 0.28–0.91), compared to the AI's index of 0.41 (95% CI: 0.064–0.71).

**Figure 3:**
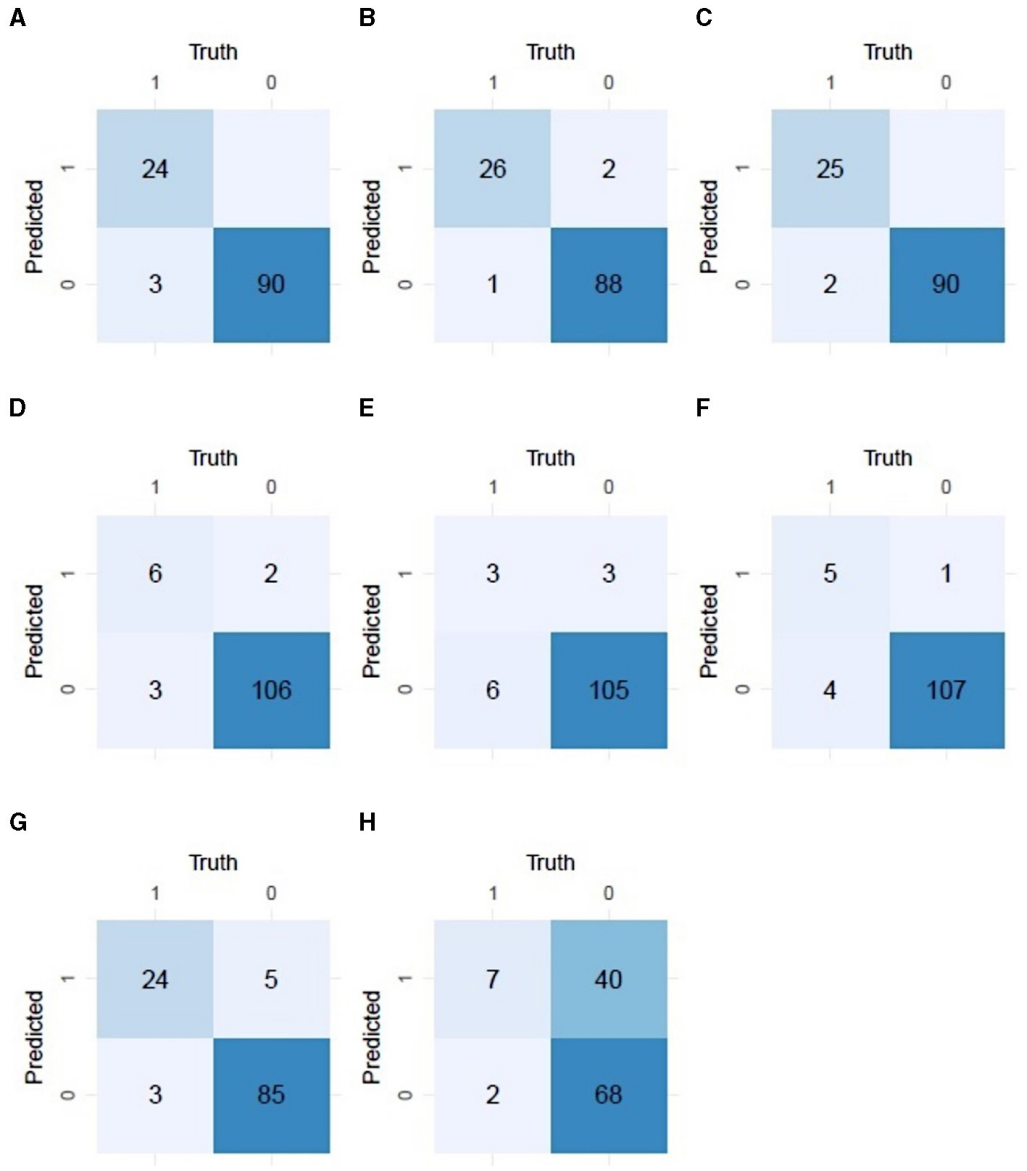
The confusion matrices for **(A–C)** melanoma nodal metastasis detection, **(D–F)** intranodal nevus detection for the three dermatopathologists, **(G)** melanoma nodal metastasis detection, and **(H)** intranodal nevus detection for the AI model.

The AI model outperformed one of the dermatopathologists and performed on par with the other two in detecting INN, although its specificity was lower than for all three dermatopathologists. The confusion matrices for NM and INN detection by the three dermatopathologists and the AI model are shown in Figure 3.

**Figure 4:**
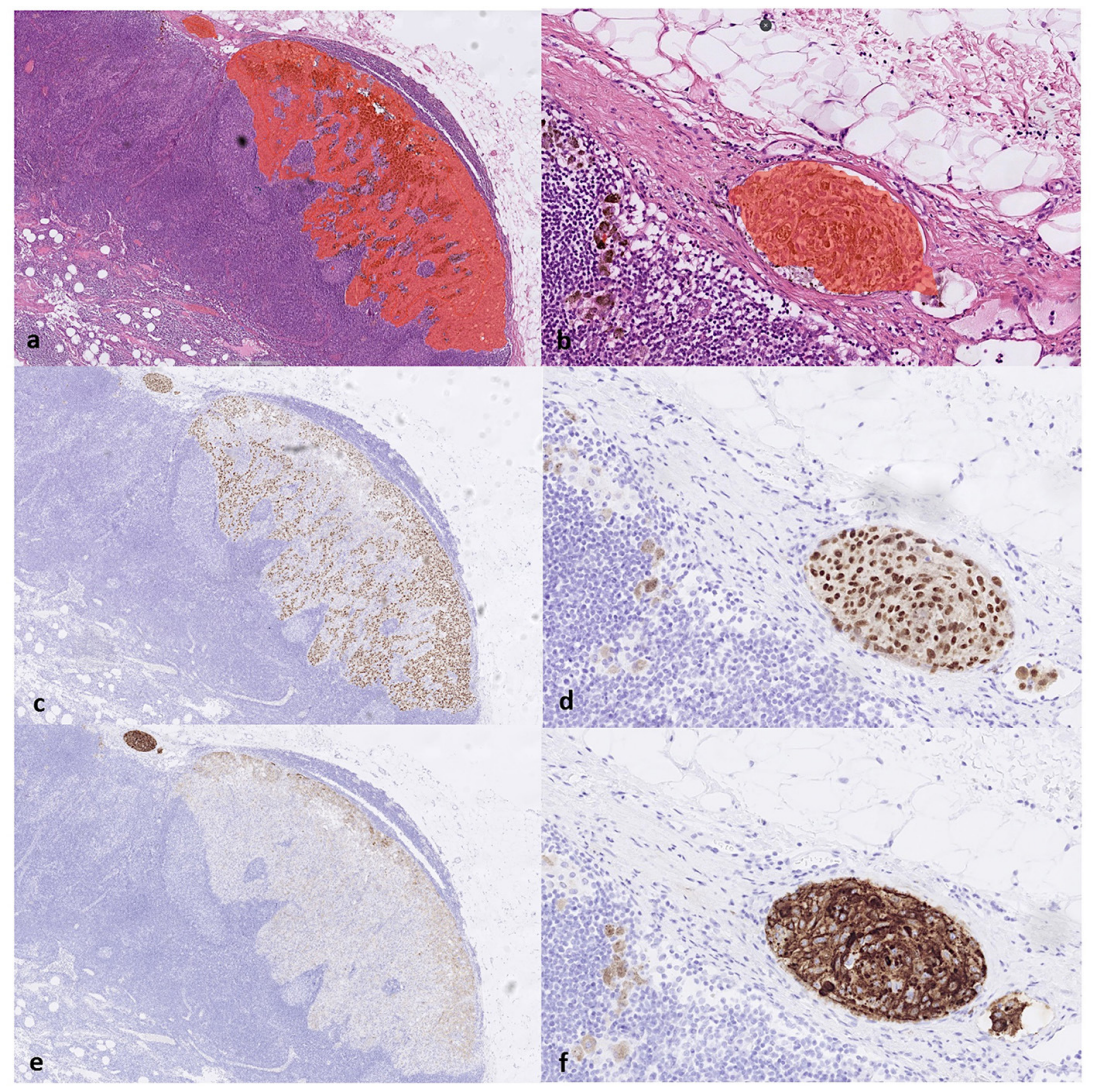
**(a)** The true positive results from the AI model correctly detect the melanoma metastasis in lymph node parenchyma and **(b)** in a lymph node capsule, as shown on H&E slides. This is confirmed by the immunohistochemistry staining serving as a reference standard. Corresponding areas stained positive for **(c, d)** SOX10 and **(e, f)** HMB45, respectively, confirming the diagnosis of melanoma metastasis (true positive).

**Figure 5:**
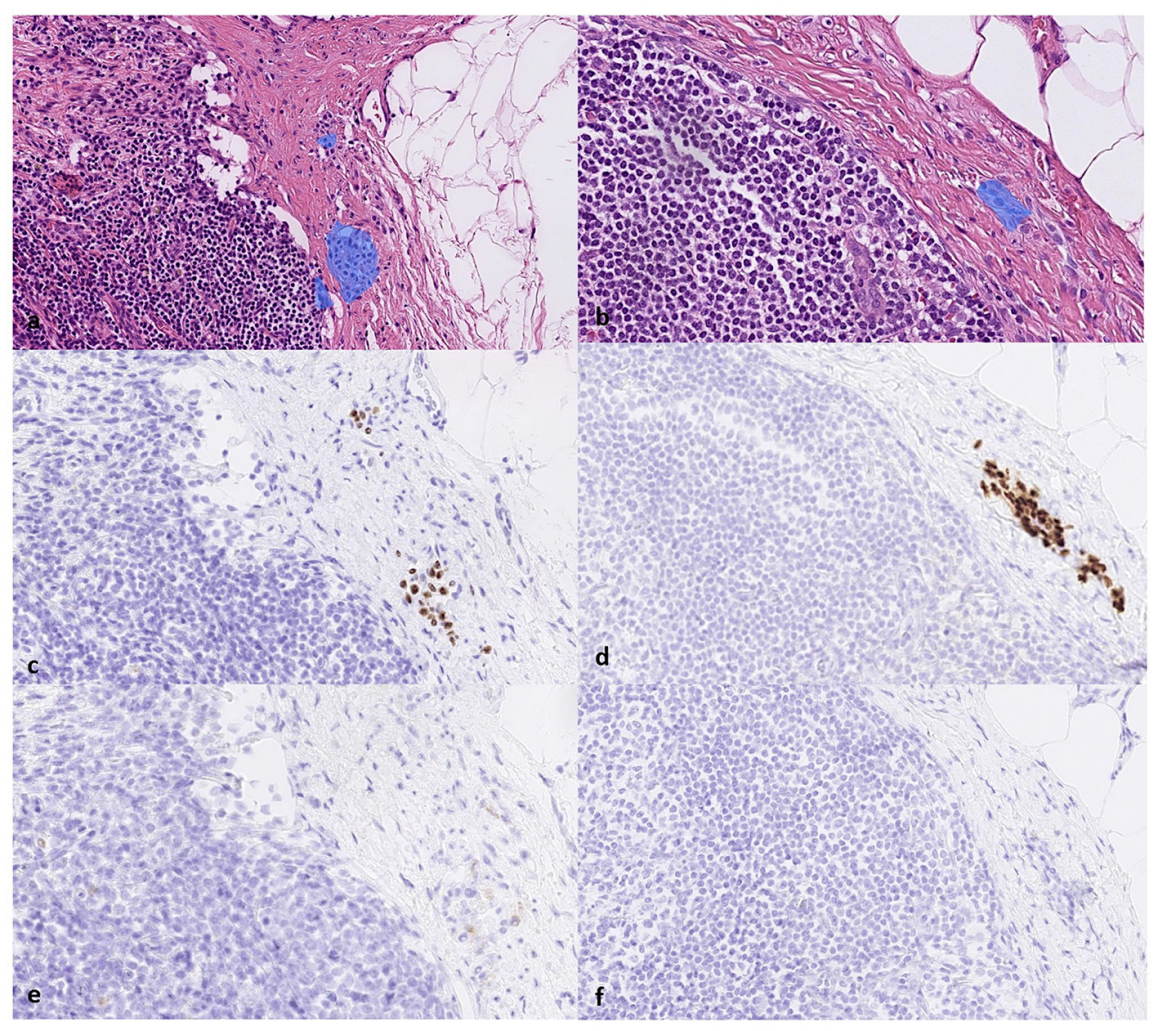
True positive results from the AI in detecting intracapsular nodal nevi on **(a, b)** H&E-slides, **(c, d)** the corresponding positive area stained for SOX10, and **(e, f)** the negative area on HMB45.

Figures 4, 5 show examples of true positive NM and INN predictions, respectively. Supplementary Figure S1 shows true positive AI predictions for NM and INN that were missed by dermatopathologists.

**Figure 6:**
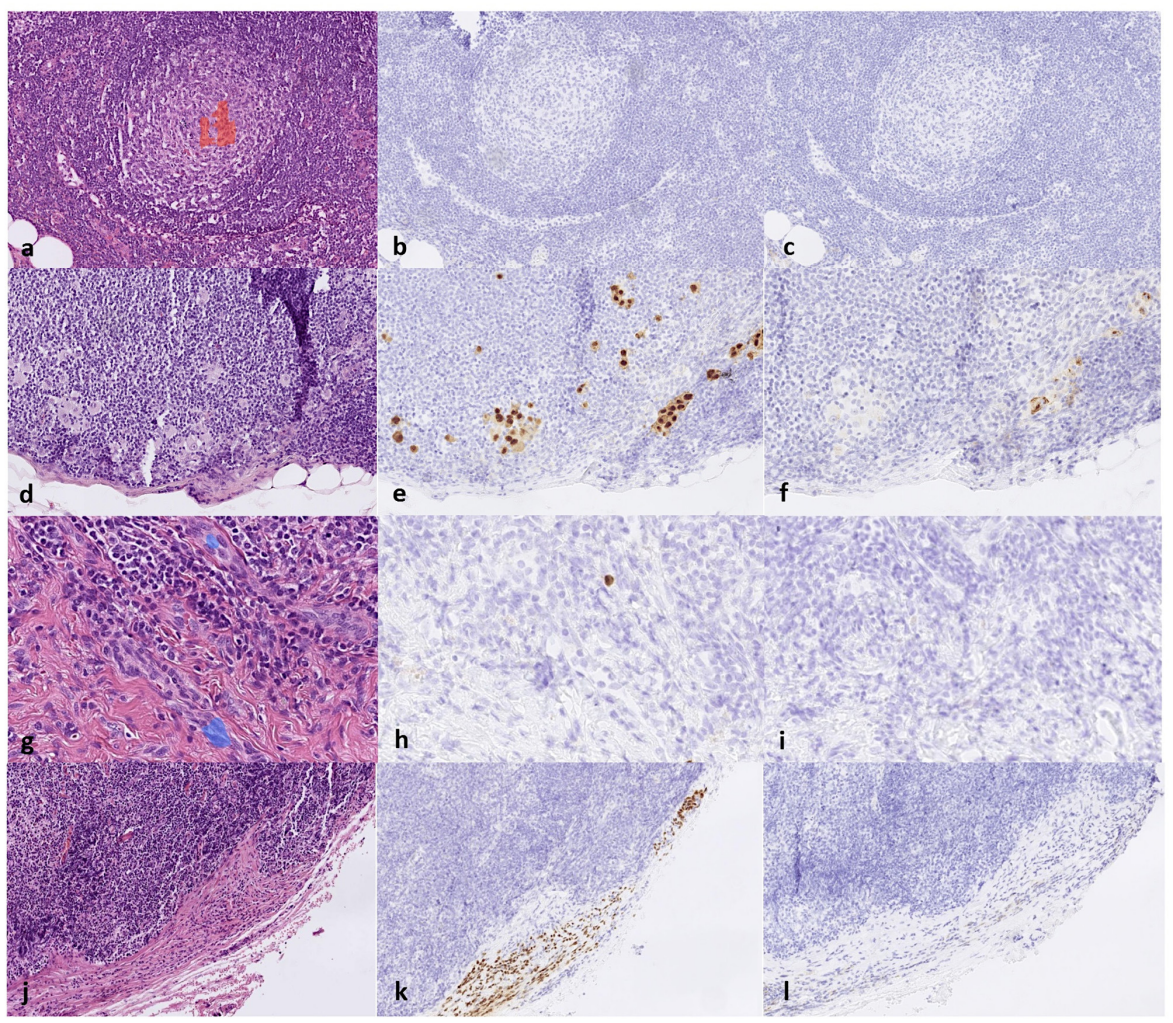
**(a)** False positive results from the AI detecting melanoma metastasis in a germinal center of the lymph node on an H&E slide and the corresponding area stained negative for **(b)** SOX10 and **(c)** HMB45; **(d)** false negative melanoma metastasis detection on an H&E slide where a small cluster of melanoma cells was interpreted as background tissue and the corresponding area stained positive for **(e)** SOX10 and **(f)** HMB45; **(g)** false positive results from the AI detecting intranodal nevus on blood vessels on an H&E slide and the corresponding area stained negative for **(h)** SOX10 and **(i)** HMB45 and, lastly, **(j)** false negative nevus detection on an H&E slide and the corresponding area stained positive for **(k)** SOX10 and **(l)** negative for HMB45.

False positive and false negative predictions are shown in Figure 6, Supplementary Figure S2.

Among the three dermatopathologists, a mean of 2.8% (95% CI: 1.4%−5.2%) of all slides was associated with a moderate or very uncertain confidence level.

From the reference standard IHC slides of the test set, the largest NM diameter per slide ranged from 0.1 mm to 21.8 mm, with a median diameter of 0.7 mm. The INN diameter ranged from 0.1 mm to 2.0 mm, with a median diameter of 0.3 mm.

## 4 Discussion

The accurate assessment of SNBs is crucial for the prognostic assessment and treatment of melanoma patients. The deep learning AI model showed excellent dermatopathologist-level performance in detecting NM and showed potential in detecting INN.

To the best of our knowledge, no previously published studies have addressed AI detection of both NM and INN. In a recently published study by Jansen et al., a deep-learning algorithm detected NM in SNBs with AUCs of 0.9630 and 0.9856 on two test cohorts from different laboratories (28). The model was trained with 542 WSIs and tested with 151 WSIs. A notable strength of the study was the collection of material from different centers and the use of various scanners, highlighting the approach's generalization capability. Additionally, the algorithm automatically measured metastasis size, a relevant feature that is lacking in our approach. Interestingly, the annotations were made with AI assistance and adjusted by a pathologist. The study solely focused on detecting NM and did not account for confounding factors such as INN. Unlike our study, the setup did not include a direct performance comparison between pathologists and the AI model.

Another previous study by Bejnordi et al. assessed AI in evaluating SNBs in breast cancer patients and showed high accuracy for AI in detecting micrometastases, surpassing a group of 11 pathologists. The study used data sets of 270 WSIs in the training set and 129 in the test set. Unlike our approach, Bejnordi et al. used a competition to identify the most accurate algorithm. The performance of the algorithms varied significantly in terms of AUC, ranging from 0.556 to 0.994, compared to our AUC of 0.961 for NM detection (18).

In our study, the performance of the AI model for detecting NM was excellent. However, the AI model did not surpass the performance of the three dermatopathologists (18). The accuracy of the individual dermatopathologists in detecting even very small NM aggregates without IHC was impressive. These pathologists were trained dermatopathologists who assessed melanoma SNBs daily and were familiar with digital diagnostics. However, their performance might have been lower under routine diagnostic conditions due to the time pressure. The study by Bejnordi et al. (18) demonstrated the negative effect of time constraints.

Furthermore, the AI model showed the potential to differentiate between NM and INN. However, the performance for INN detection was generally lower than that for NM. This discrepancy can be explained by the fact that the dataset included fewer INN cases compared to NM, and the INNs were often significantly smaller, resulting in fewer annotated INN regions compared to the NM regions. Moreover, INN cells have small, uniform nuclei without atypia, making them resemble surrounding cells, such as inflammatory cells and fibroblasts, thus posing a detection challenge compared to the atypically large NM cells.

We believe that increasing the sample size would improve the model's performance. Some INN are small, spindle-shaped, and sometimes loosely admixed in the capsule, making them easily overlooked even by experienced pathologists (8), as shown in our study. The AI model showed promise in identifying INN. However, false positives were also observed, reducing specificity (Figure 6, Supplementary Figure S2). We believe that further training could help the algorithm overcome this challenge.

We used IHC as a reference standard, along with cell morphology. Commonly used IHC stainings, such as S100, SOX10, and Melan A, cannot distinguish INN from NM (6). HMB45 staining is often negative in INN but positive in NM (8), making it useful when combined with another melanocytic marker (SOX10, S100) (29). However, the loss of HMB45 expression can occur in nodal melanoma metastases (30). In our study, all NM cases showed positivity for HMB45. Other IHC stainings that help distinguish NM from INN include PRAME, p16, and Ki67 (8, 31, 32). We used a routine staining protocol at our laboratory for MM SNBs, consisting of consecutive slides of H&E, SOX10, HMB45, and H&E. The material was retrospectively collected from the archives, and no extra staining was performed for the study.

The need for several IHC stains makes interpreting melanoma SNBs time-consuming. Despite the use of IHC, the architectural and cytologic morphology remains crucial for interpreting NM (33). The AI model could learn the morphological differences between NM and INN without IHC. INNs are often located in the capsule of the lymph nodes, while NMs are more often found in the parenchyma. Our study included NMs with various locations, including capsular foci (Supplementary Table S2). Interestingly, the algorithm could differentiate NM and INN in similar locations (Supplementary Figure 4). This supports the fact that the network can detect tumor cells regardless of the appearance of the surrounding cells or tissue. Moreover, the AI model learned to differentiate melanocytes from the surrounding pigmented macrophages.

The TNM staging system for melanoma does not include a lower threshold for the metastasis size determined in an SNB. It has been shown that melanoma patients with isolated tumor cells found in SNBs have a significantly higher risk of melanoma-specific death than those with tumor-negative SNBs (5). Interestingly, the AI model could detect even small tumor foci missed by pathologists (Supplementary Figure S1).

The method used to train the AI model was supervised deep learning, which requires extensive annotations. In our study, nearly 6,000 pixel-wise annotations were provided. To mitigate this labor-intensive process, new methods such as transfer learning and weakly supervised learning have been proposed (34, 35). One interesting method of reducing the annotation workload is the use of automated computer-assisted annotations generated by AI (28, 36).

The main strength of our study is that we included INN as a common confounding factor in the training and did not solely focus on NM. Another strength is that the dermatopathologists and the AI model assessed the same H&E-stained WSIs. We used IHC as the reference standard instead of only comparing the AI model's performance to the pathologist's assessment or previous pathology reports.

The sample size of the study can be observed as relatively small. However, the sample size was comparable to previously mentioned AI studies with a similar setup (18). The dataset was imbalanced, which could be explained by the retrospective data collection and the fact that INN is a rarer finding than NM in melanoma SNBs. This may have affected the accuracy of the model, especially in detecting INN. We collected all the cases found in the archives within the studied period. After scanning, a small number of cases were excluded due to issues with digitization, resulting in problems with image quality and focus partly due to tissue artifacts.

Moreover, some regions in the included cases showed minor artifacts (such as tissue folds and small blurry areas). Even though the AI model was trained to disregard areas with artifacts, these may have affected the performance if they were located in critical areas of the small tumor foci. Some slides were rescanned to address some of these issues, which resolved some but not all of them. Due to the limited number of cases, not all slides with minor artifacts were excluded. With increased scanning experience, issues such as blurry images could be better avoided.

The reference standard was based on a single pathologist's interpretation of IHC and H&E slides. A consensus of several pathologist interpretations would have made the reference standard more reliable. Another limitation is that we only used material from one pathology unit and did not validate the algorithm with external material from other laboratories. External validation would better evaluate the overall usability of the AI model. We aim to validate the performance in a larger study set up with more samples from multiple laboratories. However, this was not possible in this initial study.

A further limitation in our study setup was that the tumor foci in the test set were not annotated by the pathologists due to the time-consuming process and could not be compared to the AI result. This annotation was performed during the validation phase of the study to assess the potential of the AI algorithm and the need for further training. The performance of the early AI model was promising, but the training continued to increase specificity.

In the test phase, we chose a slide-level interpretation (no tumor, NM, INN, or both) performed by the AI and the dermatopathologists to obtain a more reliable picture of the performance of the model in a real-life setting (assessing the whole slides instead of small, selected regions). Similar approaches (e.g., tumor or no tumor) have been used in other SNB studies (19, 20).

There is evidence that AI-assisted diagnostics can improve the quality, efficiency, and consistency of cancer detection and grading (37). The benefits of using our approach in a real-life setting include saving time and resources. The automated WSI analysis is fast (completed in seconds) and can be performed on H&E slides. Additionally, the AI model could reduce the need for IHC and help pathologists detect the smallest tumor foci that might otherwise be missed.

In the modern digitalized pathologist workflow, the model could pre-screen WSIs and highlight slides/sections of interest for the pathologist. A pre-screening algorithm could prioritize pathology cases, allowing positive SNBs to be analyzed first. However, further validation using larger datasets and material from different laboratories scanned with various scanners is warranted before implementing the approach in real-life settings.

To conclude, the deep learning AI model showed excellent dermatopathologist-level accuracy in detecting NM and showed potential in differentiating NM from INN based on routine H&E-stained WSIs. Further validation of the method is warranted.

## Data Availability

The datasets used and/or analyzed during the current study are available from the corresponding author on reasonable request.
