# Supplementary material for: Deep learning model shows pathologist-level detection of sentinel node metastasis of melanoma and intra-nodal nevi on whole slide images
Source: Front Med (Lausanne). 2024 Aug 22;11:1418013. doi: 10.3389/fmed.2024.1418013 (PMC11374739; doi:10.3389/fmed.2024.1418013)
Supplement: Supplementary file 1 [file Data_Sheet_1.pdf]

Supplementary material

Deep learning model shows pathologist-level detection of sentinel node metastasis of melanoma and intra-nodal nevi on whole slide images

Jan Siarov[1,2], Angelica Siarov[2], Darshan Kumar[3], John Paoli[4,5], Johan Mölne[1,2], Noora Neittaanmäki [1,2]

Correspondence: Noora Neittaanmäki noora.neittaanmaki@fimnet.fi

1 Supplementary Figures and Tables

Table S1. The number of nodal melanoma metastases with microanatomical localizations included in the training, validation and test sets. The microanatomical location was defined as the deepest growth within the lymph node.

	Nodal melanoma metastases	Intra-nodal nevus
**Training set**	**30**	**18**
Capsular	0*	13
Subcapsular sinus	5	2
Trabecular	1	3
Parenchymal	12	0
**Validation set**	**18**	**10**
Capsular	0*	9
Subcapsular sinus	5	1
Trabecular	1	0
Parenchymal	12	0
**Test set**	**15**	**6**
Capsular	0*	5
Subcapsular sinus	3	0
Trabecular	3	0
Parenchymal	9	1
**Total**	**63**	**24**

*In addition to parenchymal growth, small capsular tumor foci of NM were detected in 3 cases in the training set, 3 cases in the validation set and 4 cases in the test set.

Table S2. Deep learning model hyperparameters. Convolutional neural network 1 (CNN1) i.e. tissue layer is a parent layer and CNN2 i.e. tumor layer is a child layer. They are independent layers connected to each other by filtering.

		**CNN1: Tissue**	**CNN2: Tumor**
**Classes**		Tissue	Nodal melanoma metastases,<br>Intra-nodal nevus
**Type (semantic segmentation)**		Region	Region
**Complexity***		Complex	Extra Complex
**Field of View**		200 µm	45 µm
**Training parameters**	**Weight decay**	0.0001	0.0001
	**Mini-batch size**	80	20
	**Mini-batches per iteration**	20	20
	**Iterations Without progress**	500	500
	**Initial learning rate**	0.1	1
**Image augmentation**	**Scale (min/max)**	-40/40	-10/10
	**Aspect ratio**	5	1
	**Maximum shear**	5	1
	**Luminance (min/max)**	-20/20	-10/10
	**Contrast (min/max)**	-20/20	-10/10
	**Max white balance change**	5	1
	**Noise**	1	0

* Complexity here is defined by a set number of layers and neurons per layer. The higher the complexity the higher the number of layers and the higher the number of neuros there are in this CNN-based AI outcome. All the hyperparameters mostly reflect towards changes to the image augmentation module. This image augmentation is based on the proprietary engine, enabling the desired outcome. Handling the computational burden relies on the computational requirements for the AI engines spanning over multiple redundant and non-redundant virtual servers in this case of the specific commercial cloud vendors.

Figure S1. a) The true positive results from the AI-model correctly detecting the intra-nodal melanoma metastasis missed by all pathologists, (b) intra-nodal nevus on H&E-slides missed by one dermatopathologist, c-d) corresponding areas stained positive for SOX10. The melanoma metastasis stained positive for HMB45 (e) while the intra-nodal nevus was negative for HMB45 (f) confirming the diagnoses.

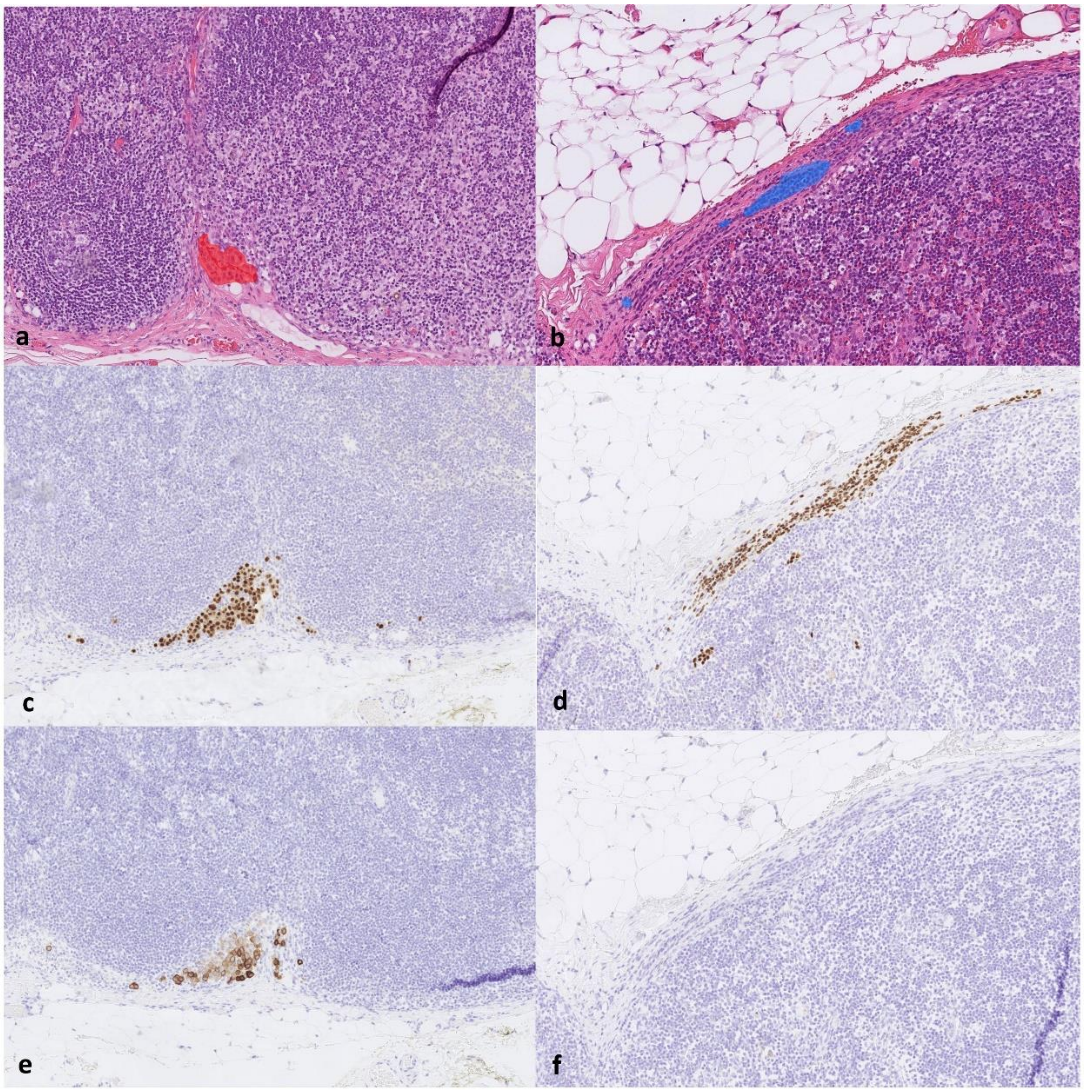

Figure S2. False positive detection by the AI model. a-b) H&E slides, c) AI misinterpreting sinus histiocytes as melanoma metastases in a germinal center of the lymph node, c) false positive nevus detection, e-f) the corresponding areas stained negative for SOX10.

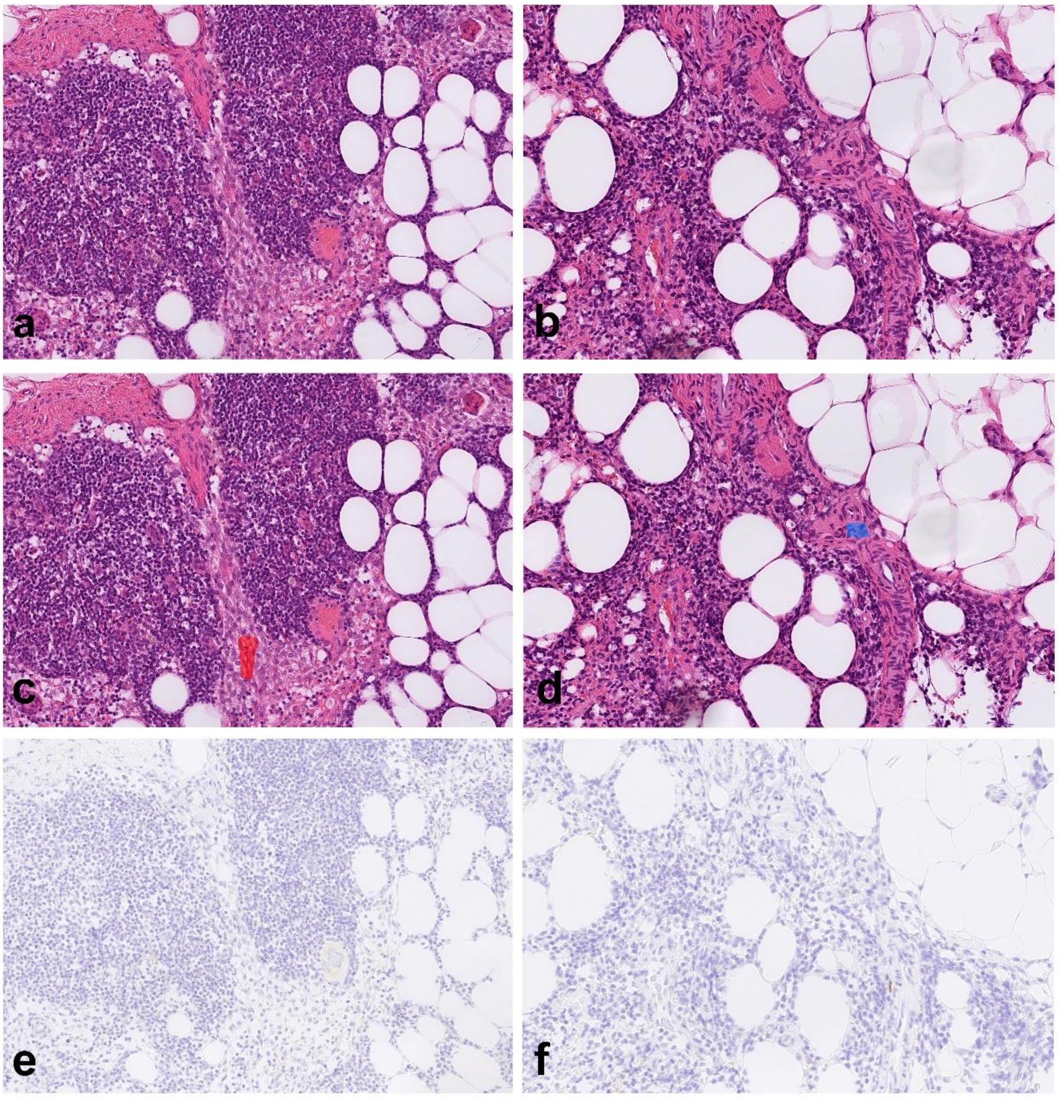

Validating the AI model

The validation of the potential performance of the algorithm was done after the first 1,046 manual annotations. A total 89 WSIs (20 with NM, 9 with NN, 2 with both NM and NN and 58 tumor-free lymph nodes) were used. Pixel-level validation was performed using the Aiforia Validation tool. The focus of the validation was to test the early performance and potential accuracy of the developed AI algorithm on unannotated H&E slides from the same laboratory. The validation regions were marked by an experienced dermatopathologist. The validation regions were approximately 100 µm in diameter and included background tissue (lymphatic tissue, connective tissue), NM and INN. A total of 272 validation regions were created including 41 regions with NM, 12 areas with INN and 219 non-tumorous. The validation regions were assessed by the AI and two dermatopathologists. Both validators, independently from each other and blinded without knowing the original pathology reports, reviewed and annotated the validation regions. The objective of the

validator was to determine if validation regions contained either NM, INN or normal tissue. The AI made the decision based solely on H&E slides while the validators had access to IHC (SOX10, HMB45) on the same cases. After validation, the AI model was further trained before running the analysis on the separate test set.

Among the two validators, the interobserver concordance was almost perfect [$\kappa = 0.90$, 95% CI 0.83–0.96). The sensitivity for predicting NM ranged from 83% to 98% for the validators compared to 88% for the early AI-model ($p=0.22$ and $p=0.73$ for validator one and validator two, respectively). The specificity ranged from 99.6% to 100% for the validators compared to 88% for the AI ($p<0.005$ and $p<0.005$). The sensitivity for predicting INN was 100% for both validators compared to 75% for the AI-model ($p=0.25$ against both validators). The specificity ranged from 98.5% to 100% for the validators compared to 92% for the AI ($p<0.005$ against both validators). After the validation we continued training the AI model to further enhance the performance. The further training included additional 4,919 annotations on the training set images and new training rounds.
